# Rapid Evaluation Methods for Quality of Trout (*Oncorhynchus mykiss*) Fresh Fillet Preserved in an Active Edible Coating

**DOI:** 10.3390/foods8040113

**Published:** 2019-04-01

**Authors:** Maria Grazia Volpe, Elena Coccia, Francesco Siano, Michele Di Stasio, Marina Paolucci

**Affiliations:** 1 Institute of Food Sciences—National Research Council (CNR), Via Roma 64, 83100 Avellino, Italy; mgvolpe@isa.cnr.it (M.G.V.); francesco.siano@isa.cnr.it (F.S.); michele.distasio@isa.cnr.it (M.D.S.); 2 Department of Sciences and Technologies, University of Sannio, Via Port’Arsa, 11, 82100 Benevento, Italy; elena.coccia@unisannio.it

**Keywords:** trout fillets, active carrageenan coating, oxidation stability, muscle structure, electronic nose, ATR-FTIR

## Abstract

In this study different methods were used to evaluate the effectiveness of a carrageenan coating and carrageenan coating incorporating lemon essential oil (ELO) in preserving the physicochemical and olfactory characteristics of trout fillets stored at 4 °C up to 12 days. The fillet morphological structure was analyzed by histological and immunological methods; lipid peroxidation was performed with the peroxide and thiobarbituric acid reactive substances (TBARS) tests. At the same time, two less time-consuming methods, such as Attenuated Total Reflectance-Fourier Transformed Infrared (ATR-FTIR) spectroscopy and the electronic nose, were used. Uncoated trout fillets (UTF) showed a less compact tissue structure than carrageenan-coated threads (CTF) and coated fillets of carrageenan (active) ELO (ACTF), probably due to the degradation of collagen, as indicated by optical microscopy and ATR-FTIR. UTF showed greater lipid oxidation compared to CTF and ACTF, as indicated by the peroxide and TBARS tests and ATR-FTIR spectroscopy. The carrageenan coating containing ELO preserved the olfactory characteristics of the trout fillets better than the carrageenan coating alone, as indicated by the electronic nose analysis. This study confirms that both carrageenan and ELO containing carrageenan coatings slow down the decay of the physicochemical and olfactory characteristics of fresh trout fillets stored at 4 °C, although the latter is more effective.

## 1. Introduction

Fish is among the most perishable food commodities whose quality declines as a result of a complex mix of biochemical, chemical, physical, and microbiological phenomena. It is estimated that ~10% of the fishery and aquaculture products is lost due to degradation (FAO report, 2018). Of primary concern is the development of off-flavors and odors caused by the production of ammonia, trimethylamine, dimethylamine, and other volatile amines, whose high levels lead to undesirable organoleptic characteristics [1]. Other volatile molecules which are mainly produced during the spoilage process are hydrogen (H2) “odorless”, methane (CH4) “odorless”, ammonia (NH3) “quaint pungent”, hydrogen sulfide (H2S) “rotten eggs”, and phosphane (PH3) “rotten fish”. The Electronic Nose has been efficiently used to detect the molecules arising during decomposition of fish flesh, even when odorless [2,3]. Not less critical is the oxidation of fat, one of the most important mechanisms leading to food spoilage, causing changes in taste and odor and deterioration of muscle texture [4]. Tenderization begins in hours after death and continues during storage [5]. The structure that is mainly involved is the cytoskeleton, linked to sarcomeres and to the plasma membrane [6,7]. Proteolysis of cytoskeletal components results in myofilament degradation [8,9]. In fish, this may include degradation of titin [10], nebulin [11], and dystrophin [12]; release of α-actinin [13]; proteolysis of myosin; and delocalization of tropomyosin [11].

The development of preservation methods in order to delay such degenerative phenomena would be desirable. Several studies have investigated the preservation technologies to maintain fish flesh elasticity and firmness. High-pressure treatment has been reported to be a useful way to improve fish flash quality by decreasing the initial activity of calpain in sea bass (*Dicentrarchus labrax*) [14]. Cold storage and freezing are classic methods for fish preservation, but they do not fully inhibit the spoilage of the quality inasmuch that they cannot prevent oxidative degradation. Therefore, as a supplement to ice or refrigeration to inhibit the normal spoilage flora, vacuum packaging (VP) and modified atmosphere packaging (MAP) are used. However, such technologies increase the costs of packaging and require specially trained workers [15,16,17]. Edible films and coatings are appealing opportunities to increase the shelf-life of perishable food products [18,19]. Both films and coatings can be made of polysaccharides, proteins, and lipids alone, or in combination, and their relevance is due to the fact that they can be safely consumed [20]. Furthermore, ingredient additives, such as antioxidant substances and antimicrobial agents can be included in the formulations for edible films and coatings in order to increase the shelf-life of foods. In their review, Socaciu et al. [21] reported ~10 different biopolymers, proteins, and polysaccharides, used alone or in combination to develop coatings and films to extend the fish fillet shelf life up to 13 days. The presence of active ingredients, mainly essential oils and polyphenols, improved the coating and film performances, especially regarding the antimicrobial properties, in both marine and freshwater fish.

In a previous paper by the same authors, the effectiveness of an edible active carrageenan coating and carrageenan coating incorporating essential lemon oil in slowing down the microbial growth and preserving the fatty acid profile of fresh trout fillets stored at 4 °C, has been described [19]. In this study, we extend our investigation using a combination of methods to assess the effectiveness of edible carrageenan coatings with or without the addition of essential lemon oil to delay the decay of muscle structure, lipid peroxidation, and olfactory characteristics of rainbow trout (*Oncorhynchus mykiss*) fillets stored at 4 °C up to 12 days. All the methods used are in agreement and indicate the coating formed by carrageenan added with ELO, as the best method to lengthen the shelf-life of trout fillets. Moreover, the ATR-FTIR spectroscopy is confirmed as a useful tool to assess the trout fillet modifications in a rapid and cost-effective fashion. 

## 2. Materials and Methods

### 2.1. Trout Fillets Preparation

Rainbow trout with an average weight of 700–800 g were purchased from a local fish farmer (Di Mella, Snata Croce del Sannio, Benevento, Italy) and brought alive to the laboratory where they were decapitated, gutted, and filleted by hand. Fillets were taken from the dorsal muscles. Skinless fillets were cut into 5 × 7 cm squares of 1 cm of thickness. Twenty-five trout were sacrificed for analyses. The present study has been conducted following the procedures for fish research established by Institutional Animal Care and Use Committee [22].

### 2.2. Coating-Forming Solutions Preparation and Treatments

Food-grade carrageenan (Sigma-Aldrich Corp., St Louis, MO, USA) was used in the coating formulations. Preliminary tests carried out in this laboratory with 0.5, 1, and 2% of carrageenan were carried out for the optimal coating formulation. One percent carrageenan gave the best results in terms of gel strengthand was therefore used for the successive coating formulation. The gel strength was evaluated with the Rowerbal method [23] According to Erkan et al. [24], 1% of essential lemon oil (ELO) was used in the carrageenan coating formulation. ELO was kindly provided by SSEA—Experimental Station of Essential Oil and Citrus Products—Reggio Calabria (Italy). ELO characterization has been reported in Socaciu et al. [21]. Carrageenan solution was prepared by mixing 1 g of carrageenan with 100 mL of distilled water and stirred at a temperature of 100 °C until the mixture became clear. Before gelation (at ~30 °C) 1% of ELO was mixed with the prepared carrageenan solution and stirred thoroughly. Trout fillets were divided into three groups and underwent to the following treatment. Group 1: uncoated trout fillets (UTF). Group 2: trout fillets coated with 1% carrageenan (coated trout fillets—CTF). Group 3: trout fillets coated with 1% carrageenan containing 1% ELO (active coated trout fillets—ACTF). Fillets were then stored in a refrigerator at 0–4 °C. During the 12-day storage, samples were randomly taken every 3 days for analyses.

### 2.3. Peroxide Value Measurement

Peroxide value (PV), expressed as meq of free iodine per kg of lipids, was performed according to the method Cd 8–53 (AOCS, 1998) of the American Oil Chemists’ Society (AOCS). Briefly, 1.0 g of lipid sample was treated with 25 mL of solvent mixture (chloroform/acetic acid 2/3). After shaking the mixture, 1 mL of saturated potassium iodide solution was added. The mixture was kept in the dark for 5 min, thereafter 75 mL of distilled water was added under stirring. Starch solution (0.5 mL, 1% *w*/*v*) was added as an indicator. The peroxide value was determined by titrating the iodine liberated from the potassium iodide (standardized with 0.01 n sodium thiosulfate solution). 

### 2.4. Thiobarbituric Acid Reactive Substances (TBARS) Determination

TBARS was evaluated according to Thiansilakul et al. [25]. The ground sample (0.5 g) was homogenized with 2.5 mL of solution containing 0.375% thiobarbituric acid (*w*/*v*), 15% trichloroacetic acid (*w/v*), and 0.25 M HCl. The mixture was heated in a boiling water bath (95–100 °C) for 10 min until it turned into a pink color, cooled with running tap water and centrifuged at 3600 g at 25 °C for 20 min. The absorbance of the supernatant was measured at 532 nm. A standard curve was prepared using 1,1,3,3-tetramethoxypropane at concentrations ranging from 0 to 6 ppm. Absorbance of TBARS was measured at 532nm and TBARS were calculated and expressed as mg malonaldehyde/kg sample.

### 2.5. Histology

Samples of 1 cm in length were taken from the central area of the fillet and placed in 2-metilbutane extra pure (Acros Organics, Fair Lawn, NJ, USA) for 5 s and then frozen in liquid nitrogen. At least three fillets were analyzed for each group. Frozen samples were serially cut on a cryostat (Leica, Wetzlar, Germany) in transversal and longitudinal sections of 10 μm. The sections were placed on slides and stained with common hematoxylin–eosin histochemical dyes. Hematoxylin staining (3 min) was followed by rinsing with deionized water and Tap water (to allow stain to develop). Acid ethanol was used to distain. Then, eosin staining (30 s) was carried out and was followed by rinsing with ethanol and Xylene. Coverslip slides were put in position by using Permount and allowed to dry. Slides were observed at an optic microscope (Leica DMRA2, Leica, Wetzlar, Germany) and images were acquired and photographed using a DC300F digital camera.

### 2.6. Electrophoresis and Western Blot Analysis

Proteins from trout fillets were extracted with Lysis buffer (220 mM D-Mannitol, 70 mM Saccharose, 1 mM EDTA, 20 mMTris, pH 7.4, containing protein inhibitors 2 mM PMSF, 1 mM pepstatin A, 2 mM trypsin inhibitor from chicken egg white). Fillets were homogenized with ultra-turraxT25 (IKA-labortechnik, Staufen, Germany) for three times at 500 rpm, 800 rpm, and 14,000 rpm for 10 min/each. The supernatants were collected and underwent protein determination with the Bio-Rad dye protein assay (Bio-Rad laboratories Inc., Perth, UK). Samples were boiled at 98 °C for 10 min in loading buffer (50 mM trisHCl pH 6.8, 100 mM β-mercaptaethanol, 2% SDS, 0.1% blue bromophenol, 10% glycerol). The proteins were separated on a 8% SDS-polyacrylamide gel electrophoresis with 4% stacking gel in 1% Tris-glycine buffer (0.025 M Tris, 0.192 M glycine and 0.1% SDS, pH 8.3) in a miniprotean cell (Bio-Rad) at 130 volts for 2 h. The separated proteins were electro transferred onto a nitrocellulose membrane with transfer buffer (39 mMTris base, 0.2 M glycine, and 20% methanol, pH 8.5), in a minitransfer cell (Bio-Rad) at 100 volts at 4 °C for 2 h. Membranes were incubated at 4°C for 1 h in blocking buffer containing 1% PBS, 0.05% Tween 20, and 5% dried, nonfat milk with antibodies raised against domain III/IV of titin (N-terminal/Z-domain) (MABT75; Millipore, Billerica, MA, USA) and polyclonal antibodies against β-actin (A5060, Sigma-Aldrich Corp., St Louis, MO, USA) as internal marker, overnight at 4 °C. Primary antibodies were diluted 1:500. The incubation with the secondary anti-mouse and anti-rabbit IgG (1:3000) was carried out for 1h at RT. Signals were detected by chemiluminescence with the Immobilon Western Chemiluminescent HRP substrate Kit (Millipore, Billerica, MA, USA) with Chemidoc (Bio-Rad). A prestained molecular weight ladder (Novex Sharp protein standard, LC5800, Invitrogen, Hilden, Germany) was used to determine the protein size. Western blotting bands were quantified by Quantity One (Bio-Rad) software version 4.6.1. 

### 2.7. Electronic Nose (EN) Analysis

The EN (PEN 3), including the Win Muster software for data analysis, Airsense Analytics Inc. (Schwerin, Germany), was used to analyze olfactory characteristics of trout fillets as previously reported [26]. For sample withdrawal, the coating was gently removed except for uncoated samples, and three cube-shaped pieces of 1g were placed in airtight 45-mL glass vial right before analysis.

### 2.8. ATR-FTIR Spectroscopy

Trout fillets were frozen, lyophilized, minced, and placed directly on the germanium piece of the infrared spectrometer with constant pressure applied. In the case of coated trout fillets, the coating was removed before the lyophilization. The pressure of the ATR-FTIR acquisition was 80 ± 2 psi. The FTIR spectra were recorded in the mid-IR region (4000–650 cm^−1^) at resolutions of 4 cm^−1^ with 32 scans using Perkin Elmer FTIR Frontier coupled with DTGS (deuterated tri-glycine sulphate) detector (Perkin-Elmer Inc., Norwalk, CT, USA). Air background spectrum was recorded before each sample. Three samples for each group were analyzed and each sample was analyzed in triplicate. The spectra were baseline corrected and normalized on amide I. 

### 2.9. Statistical Analysis

Values were expressed as mean ± standard deviation (SD) calculated using MS Excel. One-way repeated measures analysis of variance (ANOVA) was used to estimate significant differences (*p* < 0.05) during storage. All statistical analyses were performed using the STATISTICA 10.0 statistical package (Statsoft inc., Tulsa, OK, USA). To isolate the group or groups that differ from the others Multiple Comparisons versus Control Group (Holm–Sidak method) was used. For Electronic Nose, six independent measures were performed for each sample, with *n* = 3. Correlation Matrix (CM) of data was performed by using the Win Muster software. CM shows the quantitative assessment of classes severability. Values of discrimination index (DI) were in the range of 0 and 1. Values lower than 0.5 show poor severability of classes, while higher values indicate worthy severability of classes (Volpe et al. 2014) [27], values of DI ≥ 0.95 were considered significant.

## 3. Results and Discussion

### 3.1. Lipid Peroxidation

The Peroxide values in the uncoated and coated trout fillets are shown in Table 1. 

One-way repeated measures analysis of variance showed a significant difference between treatments (F = 13.055 with two degrees of freedom, *p* = 0.003). Multiple Comparisons versus control group (Holm–Sidak method) showed a significant difference in the comparison ACTF vs. UTF (*p* = 0.002) and CTF vs. UTF (*p* = 0.021).

Trout muscle tissue is rich in lipids, especially polyunsaturated fatty acids [19]. Among lipids, both free fatty acids and triglycerides are subject to oxidation, although the former are oxidized more readily. Considering that the lipid content of fresh trout is ~2.121 ± 0.06 g, the content of peroxide is equal to 0.8 meq/kg of lipids at time zero, while the sample peroxide values were respectively 8.6 meq for UTF, 5.3 for CTF and 4.0 for ACTF after 12 days of preservation. Peroxide values in the uncoated trout fillets ranged from 0.8 to 8.6 meq/Kg of lipids with a maximum at 9 days equal to 9.32 meq/kg.

Peroxide values in CTF fillets ranged from 0.8 to 5.3 meq/Kg of lipids, while in ACTF fillets ranged from 0.8 to 4.2 meq/Kg of lipids. Peroxide values were significantly lower in CTF and ACTF than in UTF. This outcome shows that the active coating slows down the development of lipid peroxidation in trout fillets stored at 4 °C. These results are in agreement with previous studies [28,29], reporting that chitosan coating was able to reduce the content of primary lipid oxidation products in herring fillets stored at about 4 °C.

The oxidation of free fatty acids produces unstable lipid hydroperoxide that readily decompose to shorter chain products such as aldehydes, which can be detected as TBARS [30].

The thiobarbituric acid reactive substances (TBARS) values in the uncoated and coated trout fillets are shown in Table 2. 

One-way repeated measures analysis of variance showed a significant difference between treatments (F = 12.781 with 2 degrees of freedom, *p* = 0.003). Multiple comparisons versus control group (Holm–Sidak method) showed a significant difference in the comparison ACTF vs. UTF (*p* = 0.003) and CTF vs. UTF (*p* = 0.005).

A substantial increase in TBARS was observed in UTF samples with respect to CTF and ACTF trout fillets. The higher efficacy of ACTF with respect to CTF, in slowing down the production of TBARS was probably due to the antioxidant and antimicrobial activity of ELO [19]. Thus, the incorporation of ELO into carrageenan coating improved the antioxidant and antimicrobial property of the resulting coating solution. Ahmad et al. [1] reported that the incorporation of ELO into gelatin film could strengthen the antimicrobial and antioxidative characteristics of the film, resulting in an increase of the qualities and the shelf-life of the sea bass refrigerated fillets. It has also been reported that ELO is effective as a free radical scavenger and metal chelating agent. The antioxidant properties of essential oils have been ascribed to different mechanisms: impediment of radical chain initiation, binding of transition metal ion catalysts, decomposition of peroxides, and interaction with the free radicals [31].

In the sea bream (*Sparus aurata*) and Atlantic salmon (*Salmo salar*) natural plant extracts have been successfully employed to prevent lipid oxidation [32,33]. Similarly, damaging of lipids was slowed down by natural antioxidants derived from barley husks in the Atlantic salmon [34]. In the cold smoked sardine (*Sardina pilchardus*) a coating enriched with oregano or rosemary extracts lowered the lipid oxidation rate [35].

### 3.2. Histological and Western Blot Analysis

**Figure 1:**
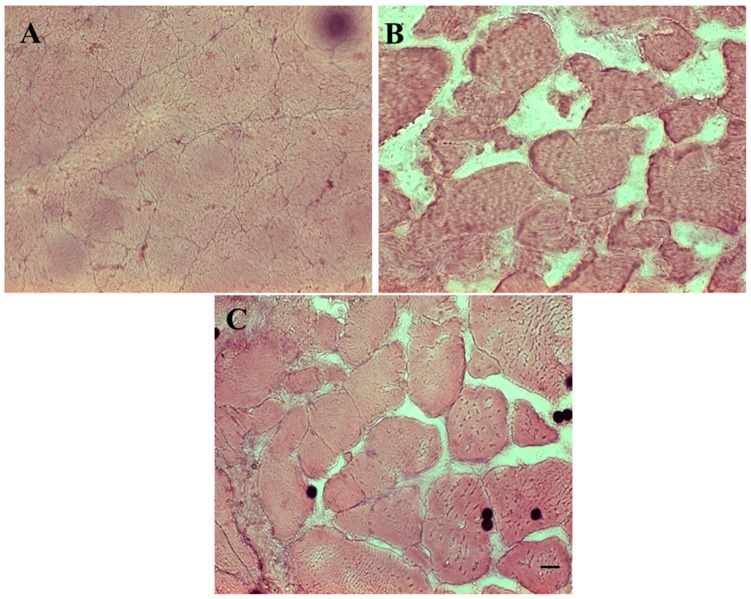
Hematoxylin–eosin staining of transversal sections of trout fillets. (**A**) uncoated trout fillet at the beginning of the experiment (UTF 0 day); (**B**) uncoated fillet after a period of 12 days of storage (UTF 12 days); (**C**) coated fillet after 12 days of storage (CTF 12 days). ACTF gave similar results to CTF. Scale bars: A, B: 40 μm; C: 20 μm. Sections are representative of three trout analyzed for each experimental group.

Fish fillets are composed of myomeres separated by connective and adipose tissues [36,37]. It has been proposed that in fish the post mortem modifications on the muscle structure and consistency are mainly due to the degradation process of the tissues between myomers rather than the muscle tissue. Ando and coworkers [5,38] demonstrated by light and electron microscopy that postmortem tenderization of rainbow trout muscle is mainly due to the disintegration of collagen fibers and the extracellular matrix in the connective tissues. In this study, both transversal and longitudinal sections of rainbow trout fillets were carried out in order to evaluate the muscle morphology. Three samples, belonging to different trout, for each treatment (UTC, CTF, and ACTF) were analyzed up to 12 days of storage at 4 °C. Transversal sections showed a compact structure with the muscle fibers firmly associated with the connective tissue at the beginning of the experiment (Figure 1A). The progression of the storage period accompanied to the modification of the fillet structure with a consequent muscle fiber disorganization both in the control and coated (CTF and ACTF) samples. In particular, the muscle fibers gradually detached from the myocommata and the distance between myofibers increased, providing the tissue with a loose aspect. However, looseness was more pronounced in the control (Figure 1B) than in the coated samples (CTF and ACTF) (Figure 1C).

**Figure 2:**
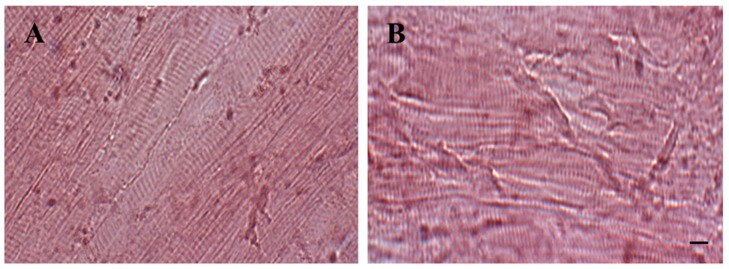
Hematoxylin–eosin staining of longitudinal sections of trout fillets. (**A**) uncoated fillet (UTF) after 12 day period of storage and (**B**) carrageenan coated fillet (CTF) after 12 days of storage; CTF and ACTF gave similar results. Scale bars: A,B: 10 μm. Sections are representative of three trout analyzed for each experimental group.

In fact, the fillet texture was more conserved during storage in the fillets with coating and coating plus ELO. Moreover, in the longitudinal sections was evident the preservation of myofibrillar structure with the alternate dark and light bands both in the control (Figure 2A) and coated samples (CTF and ACTF gave similar results) (Figure 2B). 

**Figure 3:**
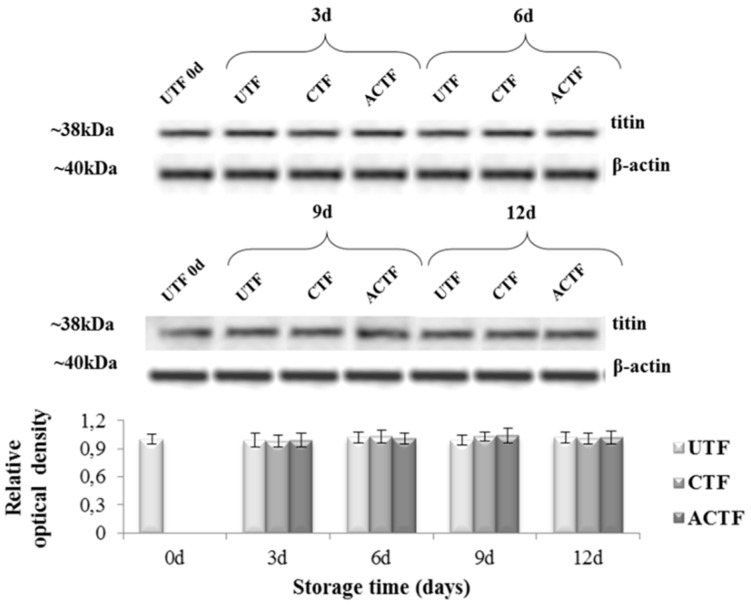
Western blotting of titin. Analysis was carried out on the control and coated fillets (UTF = uncoated fillets; CTF = coated trout fillets; ACTF = active coated trout fillets; d = day) during the storage time (0, 3, 6, 9, and 12 days). β-actin was used as an internal marker. Results are representative of three trout analyzed for each experimental group.

This outcome is sustained by the evidence that the expression of titin was stable up to 12 days of storage at 4 °C (Figure 3). 

Titin is an elastic protein, which joins the thick myosin filaments from their ends to the Z disc stabilizing the myosine in the center of the sarcomere [39]. Densitometric analysis (Figure 3) of the immunoreactive bands of titin was performed and β-actin (molecular mass of about 42 kDa), as an internal marker, was used to normalize the optical density. 

We observed neither variations of titin expression nor degradation during the storage period, both in the control and coated fillets (CTF and ACTF). Our results are in agreement with a previous study by Hernandez-Herrero et al. [40], reporting a progressive degradation of titin in the cod (*Gadus morhua*) during ice storage only when the fish was in advanced decomposition. The evidence that trout fillets coated with carrageenan and carrageenan plus ELO were well preserved suggests that the presence of the coating with or without the ELO delays the degenerative processes. Such outcome may be related to the oxygen barrier properties of edible films and coatings. Carbohydrates, such as carrageenan, are indeed excellent barriers to oxygen, because of their tightly packed, ordered hydrogen bonded network structure. Moreover, the addition of antioxidants, such as vitamins and essential oils, can entail further protection due to the enhancement of the oxygen barrier properties of the film and coating [41]. Meyer et al. [42] found that carrageenan coatings extended the shelf life of poultry pieces by acting as an oxygen barrier. Thus, it can be hypothesized that the coating used in this study protected the muscle from the oxidative processes that induce the production of free radicals, which are in turn responsible for muscle and intramuscular tissue susceptibility to proteases, with consequent postmortem tenderization of fish muscle [43]. 

### 3.3. Olfactory Analysis

**Figure 4:**
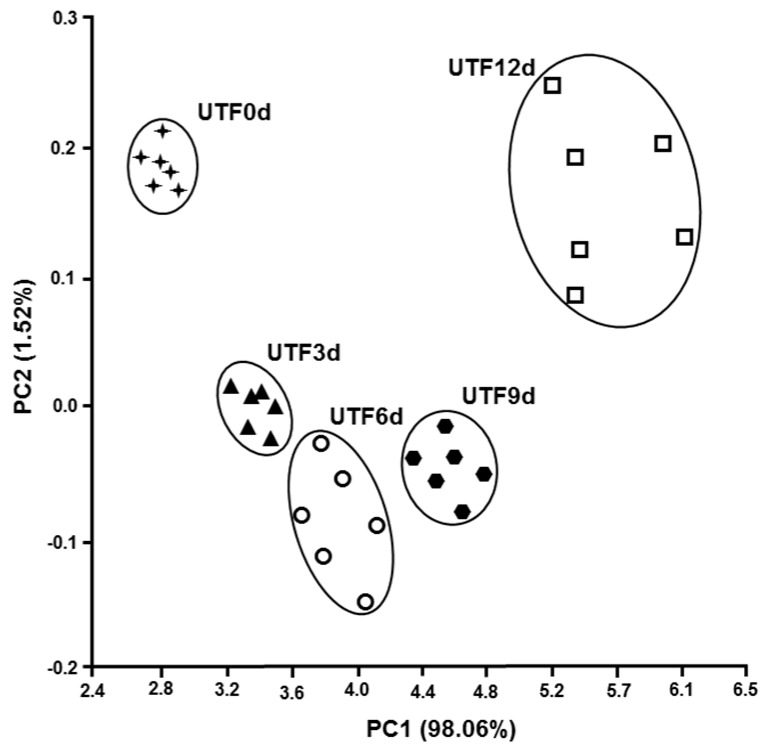
The principal components analysis (PCA) plot of the scores produced by the 10 sensor responses to the headspace of uncoated trout fillets (UTF) along the first two principal components. The number in parentheses indicates the proportion of the total variance explained by each principal component. The abbreviations near the clusters are UTF0d = uncoated trout fillet day 0 of storage; UTF3d = uncoated trout fillet day 3 of storage; UTF6d = uncoated trout fillet day 6 of storage; UTF9d = uncoated trout fillet day 9 of storage; UTF12d = uncoated trout fillet 12 days of storage. Results are representative of three trout analyzed for each experimental group.

**Figure 5:**
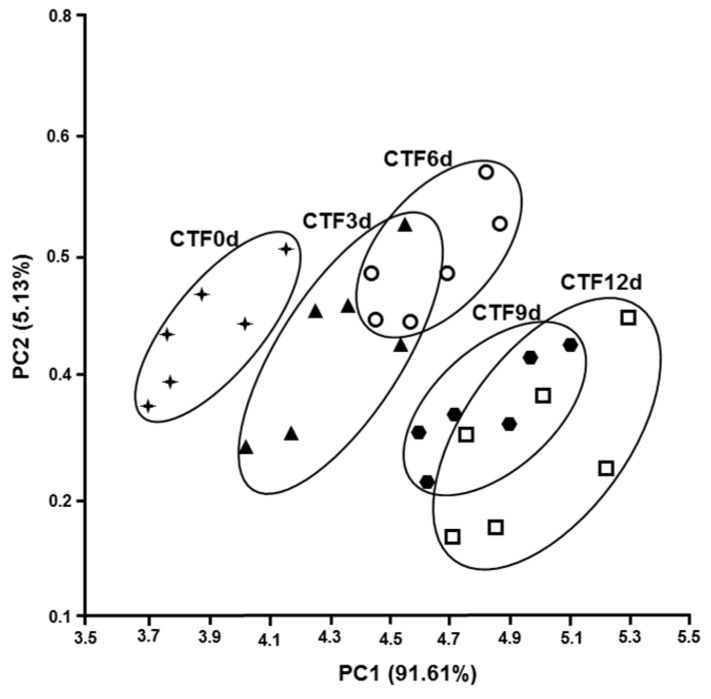
The PCA plot of the scores produced by the 10 sensor responses to the headspace of carrageenan coated trout fillets (CTF) along the first two principal components. The number in parentheses indicates the proportion of the total variance explained by each principal component. The abbreviations near the clusters are CTF0d = carrageenan coated trout fillet day 0 of storage; CTF3d = carrageenan coated trout fillet day 3 of storage; CTF6d = carrageenan coated trout fillet 6 days of storage; CTF9d = carrageenan coated trout fillet 9 days of storage; CTF12d = carrageenan coated trout fillet 12 days of storage. Results are representative of three trout analyzed for each experimental group.

**Figure 6:**
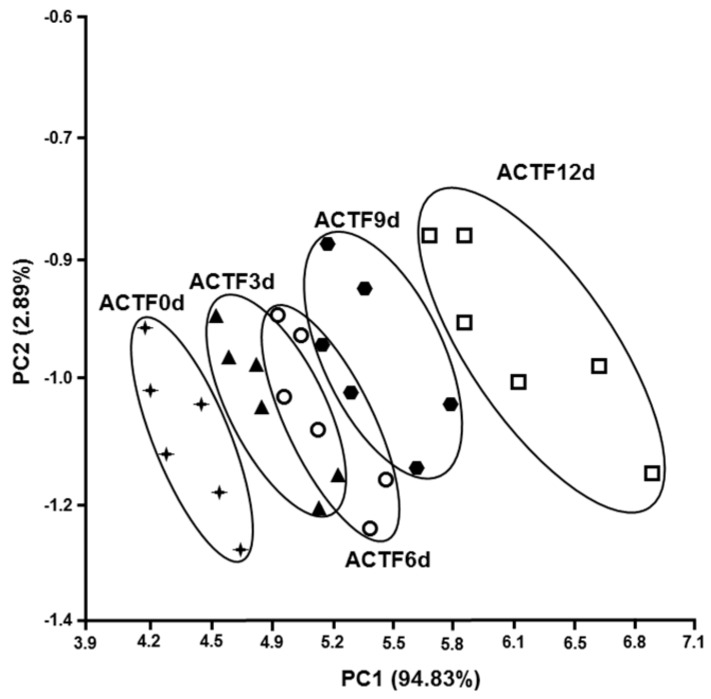
The PCA plot of the scores produced by the 10 sensor responses to the headspace of carrageenan coated trout fillets plus essential lemon oil (ACTF) along the first two principal components. The number in parentheses indicates the proportion of the total variance explained by each principal component. The abbreviations near the clusters are ACTF0d = carrageenan coated trout fillet day 0 of storage; ACTF3d = carrageenan coated trout fillet day 3 of storage; ACTF6d = carrageenan coated trout fillet 6 days of storage; ACTF9d = carrageenan coated trout fillet 9 days of storage; ACTF12d = carrageenan coated trout fillet 12 days of storage. Results are representative of three trout analyzed for each experimental group.

The smell is an important sensorial attribute of a food, not only because it is a sign of pleasantness, but also because odor, usually unpleasant, is due to microbial and biochemical alterations during food storage [44]. It is therefore important to define the olfactory characteristics of a food, as an indication of microbial and biochemical deterioration. The electronic nose is a quick and reliable method for measuring the olfactory footprint of a food. The electronic nose is made up of electronic sensors capable of detecting volatile chemicals and is able to translate these substances into a recognizable and classifiable models capable of discriminating different types of samples [45,46,47]. The relationship between the freshness of the fish and the olfactory imprint determined with the electronic nose was used by Di Natale et al. [48]. Olafsdottir et al. [49] highlighted the correlation between the olfactory footprint and bacteriological composition during the deterioration of cold-smoked Atlantic salmon. In this study, we have detected the olfactory foodprint of trout fillets stored at 4 °C up to 12 days, without and with carrageenan coating, and carrageenan coating plus ELO. Figure 4, Figure 5 and Figure 6 show the PCA of the response the 10 sensor array to the headspace of the samples. Each sample is represented by a cluster of six different measures. The processed data show a shift of the groups along the first and the second principal components, the figures also show the percentage of the variance explained for both components, with the values of the total variance being 99.58% (Figure 4), 96.74% (Figure 5), and 97.72% (Figure 6). 

It is possible to observe that all the clusters appear distinct in Figure 4. On the contrary, Figure 5 and Figure 6 show overlapping clusters corresponding to low values of discrimination indexes (Table 3, Table 4 and Table 5) between the classes observed in Table 4 and Table 5. 

The value of the first component is higher than 91.6% in the three PCA showing that the most of the variance is expressed along the x-axis. It is worth noting that the shift along x-axis of the clusters representing subsequent days of samples preservation corresponds to increasing values of discrimination indexes (DIs) of days 3, 6, 9, and 12, respective to day 0 of the correlation matrixes (CMs) (Table 3, Table 4 and Table 5). 

The DIs of the samples UTF 6d, UTF 9d, and UTF 12d correlated to UTF 0d are significant; whereas the CMs of CTF and ACTF treated samples did not show any significant value. It is possible to argue that CTF and ACTF preserved samples retained olfactory characteristics better than UTF samples during the evaluation period. The values of DIs of ACTF 3d, ACTF 6d, and ACTF 12d correlated to ACTF 0d were lower than CTF 3d; CTF 6d and CTF 12d correlated to CTF 0d.

Altogether, the data suggest that ACTF was the best gel coating formulation to preserve the olfactory characteristics of trout fillets, although samples treated with CTF formulation also showed a good performance respect to UTF samples.

### 3.4. ATR-FTIR Analysis

**Figure 7:**
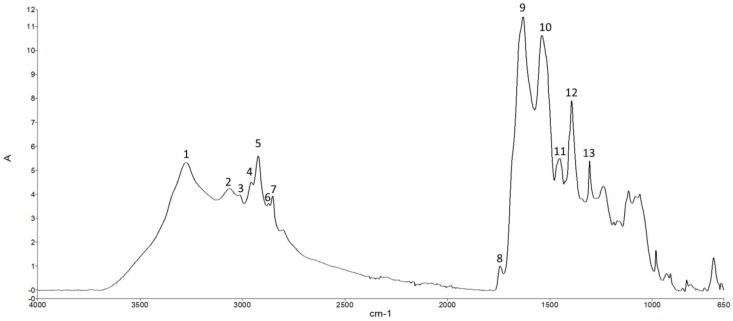
A typical ATR-FTIR absorption spectrum of the trout fillet in the 650–4000 cm^−1^. The spectrum was baseline corrected and normalized for the Amide I. The peak assignment is reported in Table 6.

The ATR-FTIR analysis was carried out in order to achieve molecular information on the biochemical modifications induced by preservation of trout fillets. Figure 7 shows a representative spectrum of trout fillet in the region of 650–4000 cm^−1^. 

The analysis of such region provides information on vibrational modes associated with the molecular composition of different functional groups belonging to lipids, proteins, and carbohydrates [50]. In this study, the contribution provided by carbohydrates was not taken into consideration due to the negligible carbohydrate content in the trout muscle tissue [51]. The peak assignment is reported in Table 6. Only the main peaks within the lipid and protein ranges are reported.

**Figure 8:**
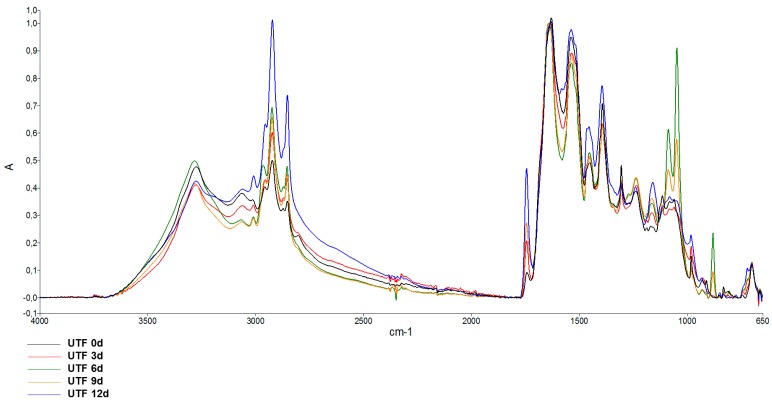
ATR-FTIR absorption spectra in the region 650–4000 cm^−1^ of uncoated (UTF) trout fillets preserved at 4 °C. Spectra were baseline corrected and normalized for Amide I. Spectra are representative of three samples.

To analyze lipid content and structure, particular attention was given to the spectral region of 2800–3100 cm^−1^ and, in particular, to the variation of the peak absorbance at 3011, 1743, 1451, and 1305 cm^−1^. The peak at 3011 cm^−1^ is usually considered a marker of peroxidative processes [57,58], and therefore its increase is indicative of a higher amount of peroxidized fatty acyl chains. In Figure 8 are reported representative spectra of the peak absorbance in UTF up to 12 days. Spectra are normalized for amide I. It can be seen that the 3011 cm^−1^ peak is slightly visible at 0 day, while it becomes more pronounced with the progression of the storage time. Similarly, the peak at 1743 cm^−1^, associated with peroxidation of fatty acid chains [59], increased over time of storage. 

**Figure 9:**
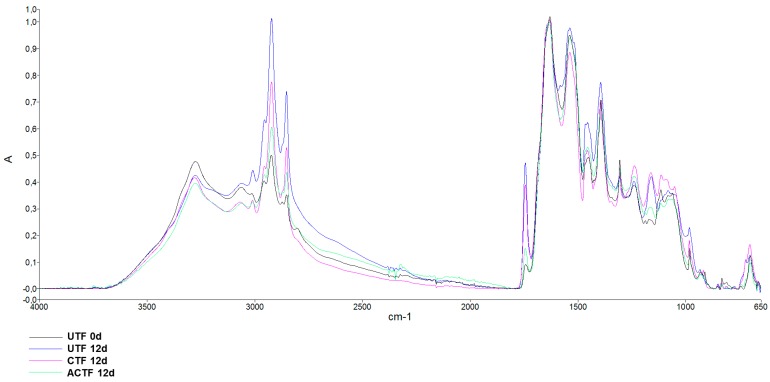
ATR-FTIR absorption spectra in the region 4000–650 cm^−1^ of uncoated (UTF), coated (CTF), and coated with ELO (ACTF) trout fillets, after 12 days of storage at 4 °C. The spectrum of trout fillet at 0 day is also shown. Spectra were baseline corrected and normalized for Amide I. Spectra are representative of three trout analyzed.

In Figure 9 are reported representative spectra of the peak absorbance of UTF, CTF, and ACTF at 12 days of storage, compared to the control at 0d.

It can be seen that the increase in the absorbance of 3011 and 1743 cm^−1^ peaks was lower in CTF and especially in ACTF with respect to UTF, suggesting that the carrageenan coating and carrageenan plus ELO coating caused the slowing down of lipid peroxidation, as also indicated by the TBARS analysis. 

**Figure 10:**
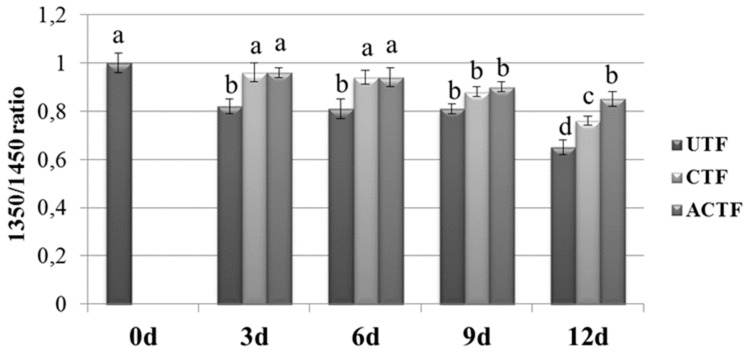
Diagram of the ratio between amide III peak (1305 cm^−1^) and the pyrrolidine peak (1451 cm^−1^) of uncoated (UTF), coated (CTF), and coated with ELO (ACTF) trout fillets, after 12 days of storage at 4 °C. Results are representative of three trout analyzed for each experimental group. Different letters on the columns indicate statistically different values (*p* < 0.05); d = day.

As reported before, the postmortem tenderization of rainbow trout muscle is likely due to the degradation of the extracellular matrix around myomers [5,38]. Collagen is the major component of the extracellular matrix and improves strength and resistance [60]. As reported by Botta et al., [56], the integrity of the collagen triple helix can be monitored by analyzing the ratio of the absorbance of the amide III and the peak corresponding to the stereochemistry of the pyrrolidine rings. The amide III band is indeed related to CN stretching and NH, and is involved with the triple helical structure of collagen [61]. The integrity of the collagen secondary structure may be verified when the value of the ratio is greater than or equal to the unit. Changes in this absorption ratio indicate significant structural alterations in the collagen triple helix. In this study, the value of the ratio between amide III peak (1305 cm^−1^) and the pyrrolidine rings peak (1451 cm^−1^) found at 0d was 1.00. The ratio decreased in UTF over time reaching a value of 0.65 after 12 days of storage. In the CTF and ACTF trout fillets the decrease in the ratio was less pronounced, reaching values of 0.76 and 0.85, respectively, after 12 days (Figure 10). 

**Figure 11:**
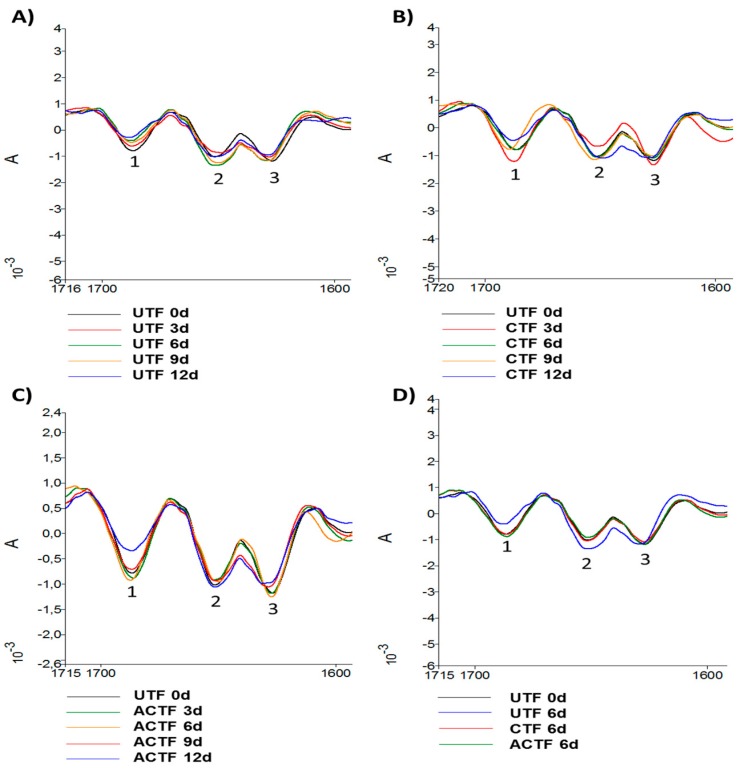
ATR-FTIR second derivative of the absorption spectra in the region 1700–1600 cm^−1^, of uncoated (UTF) (**A**), coated (CTF) (**B**), and coated with ELO (ACTF) (**C**) trout fillets, after 12 days of storage at 4 °C. The comparison of the spectrum of trout fillet at 0d and after 6 days of storage is shown in (**D**). 1 = 1628 cm^−1^ peak; 2 = 1652 cm^−1^ peak; 3 = 1687 cm^−1^ peak. Spectra are representative of three samples. d = day.

FTIR spectroscopy is a tool used to study the secondary structure of proteins [62]. In fact, the derived analysis of the amide region I, between 1600 and 1700 cm^−1^, provides information about the α and β structure of proteins [63]. In particular, vibrational components in the area of 1620–1640 cm^−1^ are indicative of a β-sheet structure. The antiparallel β-sheet structure can also be identified by the presence of vibrational components in the area of 1670–1695 cm^−1^, while α-helical conformation gives rise to infrared absorption in the range of 1650 to 1658 cm^−1^ [64]. However, it is necessary to obtain a good discrimination of the peaks in the area of the amide I through the calculation of the second derivative that amplifies the vibrational component separation [65]. The alterations in the frequency and intensity of the vibrational components or peaks are able to provide valuable information on the secondary structure and may reveal conformational changes deriving from the interaction of the protein with other molecules and with the surrounding chemical environment (pH, temperature, solvents, detergents, etc.) [63]. In this study, the second derivative of trout fillets at 0d highlights three peaks in the amide I region at the wavelengths of 1628, 1652, and 1687 cm^−1^ (Figure 11). 

The storage period of the trout fillets at 4 °C in the absence of coating shows the decrease in the intensity of the peak at 1628 cm^−1^. The peak at 1652 cm^−1^ shows an increase in intensity at 6 and 9 days, and a decrease at 3 days of storage. The 1687 cm^−1^ peak shows limited variations in intensity, while a shift from 1687 to 1685 cm^−1^ was detected. In the presence of carrageenan, there were variations in both intensity and wavelength of the peaks at 1628 and 1652 cm^−1^. In the trout fillets coated with carrageenan and ELO, the peaks at 1628 and 1652 cm^−1^ appeared, at all days of storage, very similar to trout fillets at 0 day, with the exception of the trout fillets at 12 days of storage, when both peaks appeared less intense in comparison to trout fillets at 0 day. The peak at 1687 cm^−1^ of the trout fillets at 9 and 12 days of storage showed a shift with respect to the fillets at 0 day and 3 and 6 days of storage. The comparison of the trout fillet spectra at 0 day and after 6 days of storage at 4 °C showed how in the presence of coating and coating plus ELO, the profiles were superposable to 0 day, while the profile of the untreated trout fillets clearly differed in the peak at 1628 cm^−1^ showing a decrease in intensity, in the peak at 1652 cm^−1^ showing an increase in intensity and in the peak at 1687 cm^−1^ showing a shift at 1685 cm^−1^. Variations in intensity and shift of the peaks related to the secondary structure of fish tissue proteins have been reported during the surimi gelation [66]. ATR-FTIR spectroscopy showed a significant decrease in the α-helix /β-sheet ratio in surimi after 2 years of storage at −20 °C [67]. Recently, the rearrangement of protein hydrogen bonding has been reported during surimi gelation, involving a partial change of α-helix of myosin into β-sheet, β-turn, and random coil [68]. In this study, the decrease in the 1628 cm^−1^ peak intensity during the prolonged storage at 4 °C, in both uncoated and coated trout fillets, may indicate modifications in the β-sheet structure, also confirmed by the shift of the 1687 cm^−1^ peak. The 1652 cm^−1^ peak seems to be more stable, indicating a substantial maintaining of the α-helix structure.

## 4. Conclusions

The employment of carrageenan coating and carrageenan coating enriched with ELO extended the shelf life of trout fillets stored at 4 °C. In particular, trout fillets coated with the carrageenan coating enriched with essential lemon oil are preserved better than uncoated and coated with carrageenan alone fillets. Uncoated fillets showed a more disaggregated muscle structure due to the increase of the inter muscle fiber space. The peroxide value and thiobarbituric acid reactive substances increased in coated samples more slowly than in uncoated samples during the storage period. The electronic nose analysis showed that trout fillets coated with the carrageenan coating maintained the olfactory characteristics better than the uncoated ones. All together, carrageenan coating enriched with ELO was the best to preserve the morphological, physical–chemical, and olfactory characteristics of the fresh trout fillet. The obtained results can be a major interest topic about processing and storage of a high perishable food such as fresh fish. 

## Figures and Tables

**Table 1:** Peroxide (meq kg^−1^) values in uncoated and coated trout fillets during refrigerated storage at 4 °C for 12 days *. UTF = uncoated; CTF = carrageenan coated; ACTF = carrageenan coated with ELO.

Time (Days)	UTF	CTF	ACTF
0	1.8 ± 0.02 ^A,a^	1.8 ± 0.05 ^A,a^	1.8 ± 0.07 ^A,a^
3	3.8 ± 0.06 ^A,b^	2.6 ± 0.04 ^B,b^	2.1 ± 0.06 ^C,b^
6	8.8 ± 0.04 ^A,c^	4.2 ± 0.08 ^C,c^	4.8 ± 0.03 ^B,c^
9	14.2 ± 0.07 ^A,d^	8.8 ± 0.09 ^B,d^	6.9 ± 0.09 ^C,d^
12	16.6 ± 0.02 ^A,e^	12.3 ± 0.10 ^B,e^	8.2 ± 0.03 ^C,e^

* Mean values (*n* = 3) (±SD) followed by different capital letters superindexes (A, B, C) within the same storage time and lower case letters superindexes (a, b, c, d, e) within the same column denote significant differences (*p* < 0.05).

**Table 2:** Thiobarbituric acid reactive substances (TBARS) (mg malonadehyde kg^−1^ sample) values in samples of uncoated and coated trout fillets during refrigerated storage at 4 °C for 12 days*. UTF = uncoated; CTF = carrageenan coated; ACTF = carrageenan coated with ELO.

Time (Days)	UTF	CTF	ACTF
0	0.65 ± 0.06 ^A,a^	0.65 ± 0.07 ^A,a^	0.65 ± 0.04 ^A,a^
3	0.93 ± 0.08 ^A,b^	0.72 ± 0.09 ^B,b^	0.70 ± 0.03 ^C,b^
6	1.06 ± 0.01 ^A,c^	0.84 ± 0.07 ^B,c^	0.79 ± 0.06 ^C,c^
9	1.37 ± 0.07 ^A,d^	0.90 ± 0.05 ^B,d^	0.84 ± 0.08 ^C,d^
12	1.89 ± 0.13 ^A,e^	0.98 ± 0.04 ^B,e^	0.89 ± 0.06 ^C,e^

* Mean values (*n* = 3) (±SD) followed by different capital letters superindexes (A, B, C) within the same storage time and lower case letters superindexes (a, b, c, d, e) within the same column denote significant differences (*p* < 0.05).

**Table 3:** Correlation Matrix among the uncoated trout fillets (UTF). The numbers indicate the discrimination indexes.

	UTF 0d	UTF 3d	UTF 6d	UTF 9d	UTF 12d
UTF 0d	0.000				
UTF 3d	0.940 ^a^	0.000			
UTF 6d	0.954 ^b^	0.790 ^a^	0.000		
UTF 9d	0.979 ^b^	0.946 ^a^	0.804 ^a^	0.000	
UTF 12d	0.985 ^b^	0.951 ^b^	0.911 ^a^	0.788 ^a^	0.000

0, 3, 6, and 12d indicate the preservation days. a: values ≥ 0.50 and < 0.95; b: values ≥ 0.95. Values of discrimination indexes ≥ 0.95 are significant.

**Table 4:** Correlation matrix among the carrageenan coated trout fillets (CTF). The numbers indicate the discrimination indexes.

	CTF 0d	CTF 3d	CTF 6d	CTF 9d	CTF 12d
CTF 0d	0.000				
CTF 3d	0.588 ^b^	0.000			
CTF 6d	0.786 ^b^	0.480 ^a^	0.000		
CTF 9d	0.811 ^b^	0.533 ^b^	0.611 ^b^	0.000	
CTF 12d	0.873 ^b^	0.729 ^b^	0.561 ^b^	0.247 ^a^	0.000

0, 3, 6, and 12d indicate the preservation days. a: values < 0.50; b: values ≥ 0.50 and < 0.95. Values of discrimination indexes ≥ 0.95 are significant.

**Table 5:** Correlation matrix among the carrageenan coated trout fillets plus essential lemon oil (ACTF). The numbers indicate the discrimination indexes.

	ACTF 0d	ACTF 3d	ACTF 6d	ACTF 9d	ACTF 12d
ACTF 0d	0.000				
ACTF 3d	0.490 ^a^	0.000			
ACTF 6d	0.632 ^b^	0.436 ^a^	0.000		
ACTF 9d	0.820 ^b^	0.587 ^b^	0.395 ^a^	0.000	
ACTF 12d	0.863 ^b^	0.773 ^b^	0.715 ^b^	0.544 ^b^	0.000

0, 3, 6, and 12d indicate the preservation days. a: values < 0.50; b: values ≥ 0.50 and < 0.95. Values of discrimination indexes ≥ 0.95 are significant.

**Table 6:** Assignment of ATR-FTIR peaks of trout fillets. Assignment according to Malek et al. [52], Movasaghi et al. [53], De Campos Vidal and Mello [54], Olezko et al. [55], and Botta et al. [56].

Peak Number	Peak Wavelength (cm^−1^)	Vibrational Mode	Components
1	3276	N–H stretch (Amide A)	Proteins
2	3063	N–H stretch (Amide B)	Proteins
3	3011	=CH oleficic stretch	Unsaturated lipids
4	2957	CH_3_ asymmetric stretch	Lipids and proteins
5	2923	CH_2_ asymmetric stretch.	Saturated lipids and side chains of proteins, cholesterol, phospholipids
6	2873	CH_3_ symmetric stretch	Lipids and proteins
7	2853	CH_2_ symmetric stretch.	Saturated lipids and side chains of proteins
8	1743	C=O stretch	Lipids, phospholipids
9	1630	C=O stretch + NH bend (amide I)	Proteins
10	1539	N–H bend + C–N stretch (amide II)	Proteins
11	1451	CH_2_ Scissoring vibrations	Pyrrolidine rings of proline and hydroxyproline
12	1393	COO^−^ symmetric stretch.	Fatty acids, amino acids
13	1305	Amide III	Proteins
